# Supplementary material for: MetaFetcheR: An R Package for Complete Mapping of Small-Compound Data
Source: Metabolites. 2021 Oct 28;11(11):743. doi: 10.3390/metabo11110743 (PMC8620779; doi:10.3390/metabo11110743)
Supplement: Supplementary file 1 [file metabolites-11-00743-s001.zip › metabolites-1380995-supplementary_materials.pdf]

MetaFetcheR: An R package for complete mapping of small compound data

Sara A. Yones[1,*], Rajmund Csombordi[1], Jan Komorowski[1,2,3,4], Klev Diamanti[1,5,*]

[1]Department of Cellular and Molecular Biology, Uppsala University, Uppsala, Sweden

[2]Institute of Computer Science, Polish Academy of Sciences, Warsaw, Poland

[3]Washington National Primate Research Center, Seattle, WA, USA

[4]Swedish Collegium for Advanced Study, Uppsala, Sweden

[5]Department of Immunology, Genetics and Pathology, Uppsala University, Uppsala, Sweden

* Correspondence: sara.younes@icm.uu.se; Tel.: +46-76-592-2512 or klev.diamanti@igp.uu.se; Tel: +46-73-926-748

Supplementary Material

1 Supplementary Note

1.1 Results of benchmarking mapping performance of MetaFetcheR

Performance of MetaFetcheR was benchmarked based on three case studies using two datasets and and three existing tools. The first dataset by Diamanti et al [1] and the second one by Priolo et al [2]. The three tools are MS_targeted , MetaboAnalystR along with MetaboAnalyst 5.0 web tool and Chemical Translation Service (CTS) [1,3–5]. The details of the benchmarking design and the metrics for each case study are described in the Materials and Methods section.

Case 1

We compared the performance of the algorithm for mapping metabolite identifiers to the mapped identifiers using MS_targeted along with the cases that were manually curated (that could not be mapped using MS_targeted). MS_targeted was run on Diamanti et al [1] dataset and had a higher number of unmapped identifiers (PubChem: ~99%, ChEBI: ~88%, LIPID MAPS: ~67%, KEGG: ~50% and HMDB: ~24%) compared to MetaFetcher (PubChem: ~14%, ChEBI: 14%, LIPID MAPS: ~67%, KEGG: ~32%, HMDB: ~19%). Subsequently, the results from MS_targeted were manually curated and compared to results of MetaFetcheR. There was ~80% match on mapped identifiers between MetaFetcheR and MS_targeted along with the manually curated ones that could not be mapped using MS_targeted (Supplementary Table S12 & Supplementary Figure S6). The results showed high concordance between the MetaFetcheR mapping and the manual curation of MS_targeted results which proves the superiority of MetaFetcheR over MS_targeted. Only LIPID MAPS showed less than ~80% matches between mapped identifiers for both tools. This was possibly due to the sparse input for LIPID MAPS that had only 68 entries. The used input dataset can be found in (Supplementary Table S13).

Case 2

We compared the MetaFetcheR mapping performance to that of the mapping function of MetaboAnalystR [3] using data from Diamanti et al and Priolo et al [1,2]. For the comparison in which metabolites names was used as an input to both tools MetaFetcheR outperformed MetaboAnalystR on Diamanti et al [1] dataset (Figure 2B & Supplementary Table S1). MetaFetcheR had the lowest percentage of unmapped identifiers for all four databases (HMDB: 19%, KEGG: 32%, ChEBI: 14%, PubChem: 14%) compared to the unmapped identifiers using MetaboAnalystR (HMDB: 51%, KEGG: 57%, ChEBI: 53%, PubChem: 44%). Out of the metabolites' identifiers that MetaboAnalystR was able to map (HMDB: 201 identifiers, KEGG: 178 identifiers, ChEBI: 195 identifiers, PubChem: 233 identifiers) MetaFetcheR was able to match with (HMDB: 88%, KEGG: 89%, ChEBI: 88%, PubChem: 81%) of them. MetaFetcheR had a higher coverage of mapped identifiers (HMDB: 336 identifiers, KEGG: 281 identifiers, ChEBI: 355 identifiers, PubChem: 357 identifiers). For MetaboAnalyst 5.0 web tool, it was able to map 325 out of the 414 metabolites names to metabolites identifiers and MetaFetcheR still showed competence in this case (Figure 2C, Supplementary Table S3).

As mentioned previously in the Materials and Methods section, MetaboAnalyst 5.0 can accept metabolites identifiers as input (a list of the same identifier type) except LIPID MAPS identifier. Out of the 327 HMDB metabolites identifiers available in [1] MetaboAnalyst 5.0 webtool was able to map 262 and it did not return any results for the 65 remaining metabolites data while MetaFetcheR returned results for all the 327 metabolites. Similarly, out of the 219 KEGG identifiers MetaboAnalyst 5.0 webtool was able to map 147 while MetaFetcheR returned results for all 219 metabolites. For the available 153 PubChem identifiers MetaboAnalyst 5.0 webtool was able to map 115 while MetaFetcheR returned results for all 153 identifiers.

We compared the mapping performance of MetaFetcheR to MetaboAnalystR using Priolo et al [2] dataset. There was only one metabolite name that MetaboAnalystR was not able to map which was linolenate_[alpha_or_gamma_(18:3n3_or_6)]. MetaFetcheR outperformed MetaboAnalystR on Priolo et al [2] dataset (Supplementary Table S1). MetaFetcheR had the lowest percentage of unmapped identifiers for all four databases (HMDB: 11%, KEGG: 0%, ChEBI: 4%, PubChem: 4%) compared to the unmapped identifiers using MetaboAnalystR (HMDB: 27%, KEGG: 28%, ChEBI: 25%, PubChem: 19%). Out of the metabolites that MetaboAnalystR could map (HMDB: 166 identifiers, KEGG: 163 identifiers,

ChEBI: 170 identifiers, PubChem: 183 identifiers) MetaFetcheR was able to match with (HMDB: 73%, KEGG: 96%, ChEBI: 75%, PubChem: 69%) of them. MetaFetcheR had higher coverage of mapped identifiers (HMDB: 203 identifiers, KEGG: 227 identifiers, ChEBI: 218 identifiers, PubChem: 217 identifiers) (Figure 2B & Supplementary Table S2). For MetaboAnalyst 5.0 web tool, it was able to map 184 metabolites out of the 227 metabolites names to metabolites identifiers and MetaFetcheR still showed competence in this case (Figure 2D & Supplementary Table S4). MetaboAnalyst 5.0 webtool was able to map 212 metabolites out of the available 227 KEGG identifiers in Priolo et al [2] dataset which was used as input while MetaFetcheR returned results for 220 metabolites). The dataset that was used as an input can be found in (Supplementary Table S14)

Case 3

We compared MetaFetcheR mapping performance to that of the mapping function of Chemical Translation Service CTS [5] using data from Diamanti et al and Priolo et al[1,2].

MetaFetcheR outperformed CTS on Diamanti et al [1] dataset (Supplementary Figure S4 & Supplementary Tables S15,S16 & S17). For the first run using HMDB identifiers, MetaFetcheR had the lowest percentage of unmapped identifiers for all three databases (KEGG: 20%, ChEBI: 1%, LIPID MAPS: 73%) compared to the unmapped identifiers using CTS (KEGG: 94%, ChEBI: 87%, LIPID MAPS: 92%). Out of the metabolites that CTS was able to map (KEGG: 21 identifiers, ChEBI: 44 identifiers, LIPID MAPS: 30 identifiers) MetaFetcheR was able to match with (KEGG: 91 %, ChEBI: 91%, LIPID MAPS: 67%) of them. MetaFetcheR had higher coverage of mapped identifiers (KEGG: 263 identifiers, ChEBI: 325 identifiers, LIPID MAPS: 88 identifiers). Similarly, for the second run using KEGG identifiers, MetaFetcheR had the lowest percentage of unmapped identifiers for two databases (HMDB: 17%, ChEBI: 1%) compared to the unmapped identifiers using CTS (HMDB: 19%, ChEBI: 16%). The percentage of unmapped identifiers for LIPID MAPS was similar for both MetaFetcheR and CTS in this case with 79% for the former and 78 % for the latter. Out of the metabolites' identifiers that CTS was able to map for HMDB and ChEBI (HMDB: 177 identifiers, ChEBI: 184 identifiers) MetaFetcheR was able to match with (HMDB:77%, ChEBI: 84%) of them. MetaFetcheR had higher coverage of mapped identifiers in general when HMDB and ChEBI identifiers were used as input (KEGG: 182 identifiers, ChEBI: 217 identifiers). Finally, for the last run with LIPID MAPS identifiers, MetaFetcheR had the lowest percentage of unmapped identifiers for all three databases (HMDB: 28%, KEGG: 49%, ChEBI: 12%) compared to the unmapped identifiers using CTS (HMDB: 56%, KEGG: 74%, LIPID MAPS: 49%). Out of the metabolites identifiers that CTS was able to map for all three databases (HMDB:30 identifiers, KEGG: 18 identifiers, ChEBI: 35 identifiers) MetaFetcheR was able to match with (HMDB:100%, KEGG:100%, ChEBI:89%) of them.

The same experiment was repeated on Priolo et al [2] dataset with the available KEGG identifiers (Supplementary Figure S5 & Supplementary Tables S18). MetaFetcheR had the lowest percentage of unmapped identifiers for all three databases (HMDB: 11%, ChEBI: 12%, LIPID MAPS: 82%) compared to the unmapped identifiers using CTS (HMDB: 22%, ChEBI: 14%, LIPID MAPS: 85%). Out of the metabolites identifiers that CTS was able to map for all three databases (HMDB:178 identifiers, ChEBI: 197 identifiers, LIPID MAPS: 36 identifiers) MetaFetcheR was able to match with (HMDB:87%, ChEBI:85%, LIPID MAPS:89%) of them.

2 Supplementary Figures

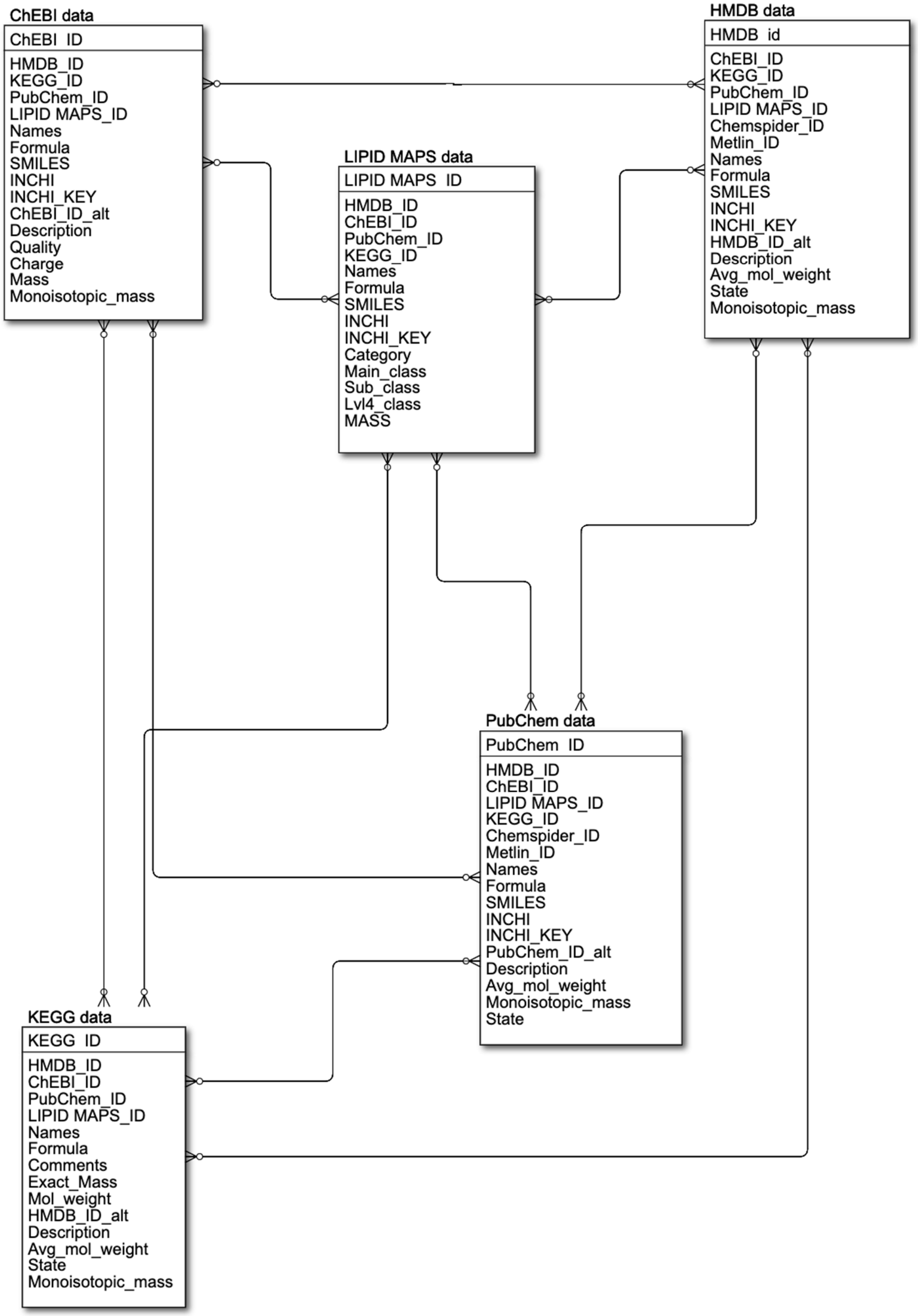

Supplementary Figure S1. Entity Relationship Diagram (ERD) of PostgreSQL database that MetaFetcheR builds locally. PK stands for primary key and FK stands for foreign keys.

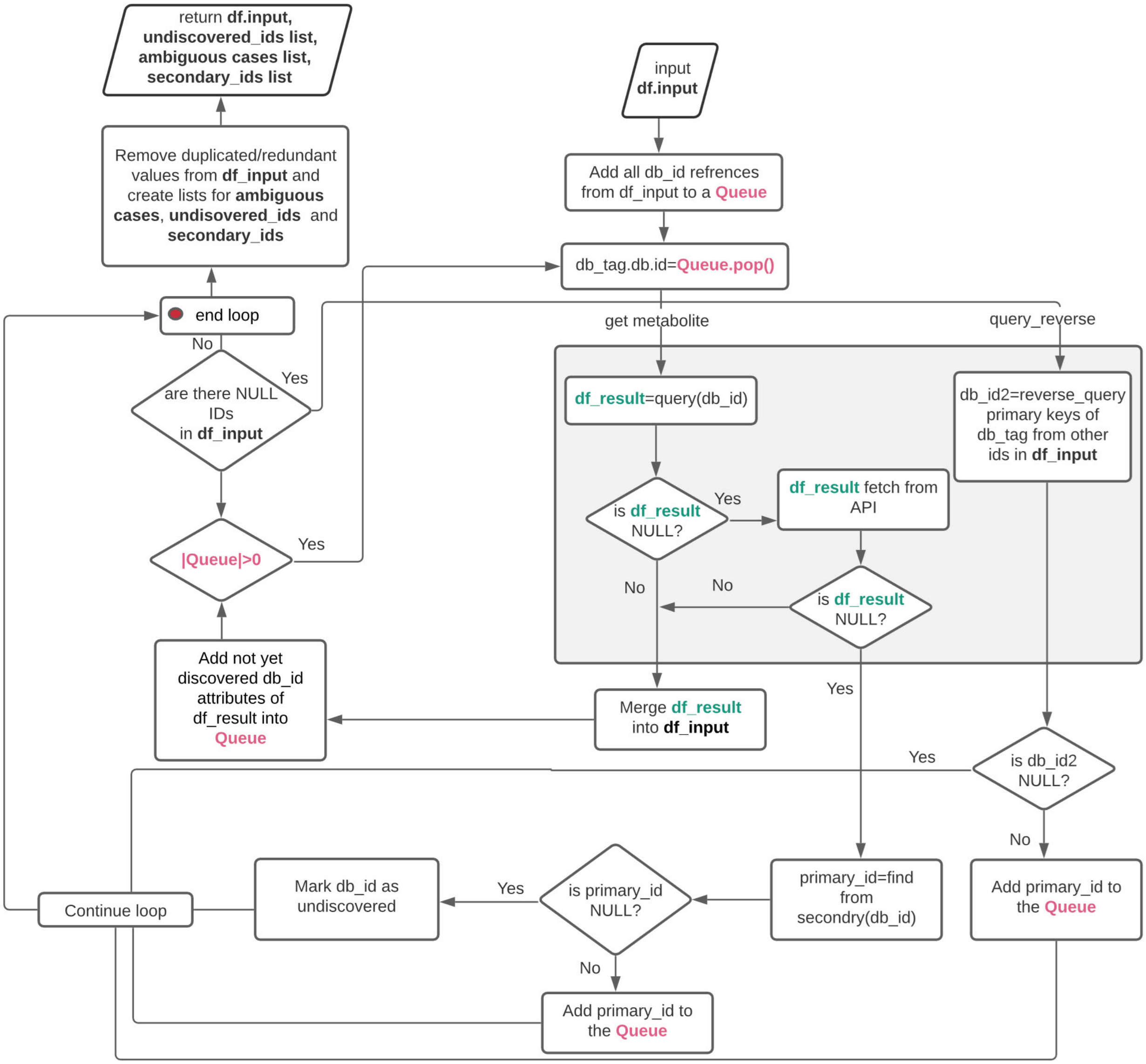

Supplementary Figure S2. Detailed flow chart of the MetaFetcheR algorithm. df.input is the initial table provided by the user and df.result is the output table. db_tag represents the name of the database, namely HMDB, ChEBI, KEGG, PubChem and LIPID MAPS, and db_id represents the id related to a certain db_tag. The queue is represented in bolded pink color, df_result, which represents the table that contains the query results, is represented in bolded green and df_input, which represents the input table, is represented in bolded black color, undiscovered_ids list which represents a list marking all possible IDs that were used for searching and returned no result, is represented by bolded black color, ambiguous cases list which represents a list of all records in the final df_input containing more than a single value for any of the IDs after the search algorithm ends, is represented in bolded black color, secondary_ids list which represents a list of all possible IDs that were used in the search algorithm as secondry_ids, is represented in bolded black color. The grey box represents the main routine, which handles querying the different databases and APIs.

A

HMDB

Metabolite Name	HMDB_ID	ChEBI_ID
Diethyl_desulfide	HMDB0029572	

ChEBI

Metabolite Name	ChEBI_ID	HMDB_ID
-	-	

B

HMDB

Metabolite Name	HMDB_ID	ChEBI_ID
ProstoglandinA1	HMDB0002656	15545

ChEBI

Metabolite Name	ChEBI_ID	HMDB_ID
ProstoglandinA1	15545	?

C

HMDB

Metabolite Name	HMDB_ID	ChEBI_ID
Fenoterol	HMDB0015405	149226

ChEBI

Metabolite Name	ChEBI_ID	HMDB_ID
Fenoterol	149227	HMDB0015405

Supplementary Figure S3. A representation of possible scenarios for mapping inconsistencies of metabolite identifiers between HMDB and ChEBI. The red color in the tables marks sample inconsistencies. **A)** The metabolite diethyl disulfide has an identifier in HMDB but does not have an entry in ChEBI. **B)** The metabolite prostaglandin A1 has an entry in HMDB with HMDB and ChEBI identifiers mapped to each other. However, the same metabolite has an entry in ChEBI but the link to HMDB is missing. **C)** The metabolite fenoterol has an entry in HMDB that is linked to ChEBI, however, the same metabolite has an entry in ChEBI with a different ChEBI identifier but the same HMDB identifier.

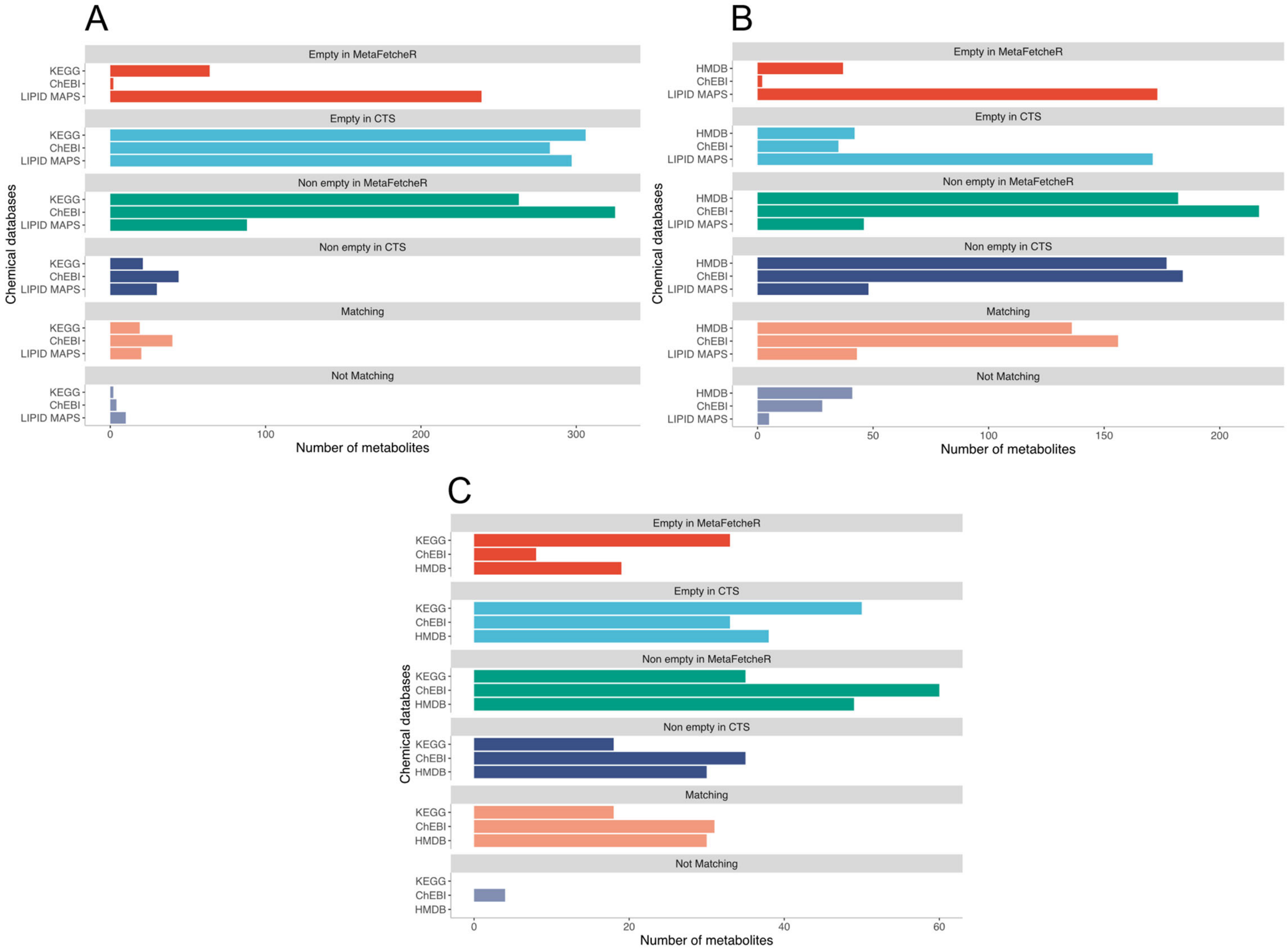

Supplementary Figure S4. Mapping performance comparison of MetaFetcheR to CTS on the dataset from Diamanti et al [1]. A)Performance comparison based on mapping HMDB identifiers available in Diamanti et al [1] dataset to other identifiers, **B)** Performance comparison based on mapping KEGG identifiers available in Diamanti et al [1] dataset to other identifiers, **C)** Performance comparison based on mapping LIPID MAPS identifiers available in Diamanti et al [1] dataset to other identifiers. Empty in MetaFetcheR and CTS panels illustrate the number of identifiers that could not be mapped using the respective tool. Non-empty in MetaFetcheR and CTS panels present the number of identifiers that were successfully mapped using the respective tool. Matching panel shows the number of mapped identifiers that agreed between tools. Non-matching panel shows the number of mapped identifiers that were not in agreement between tools. The number of identifiers is shown on the x-axis.

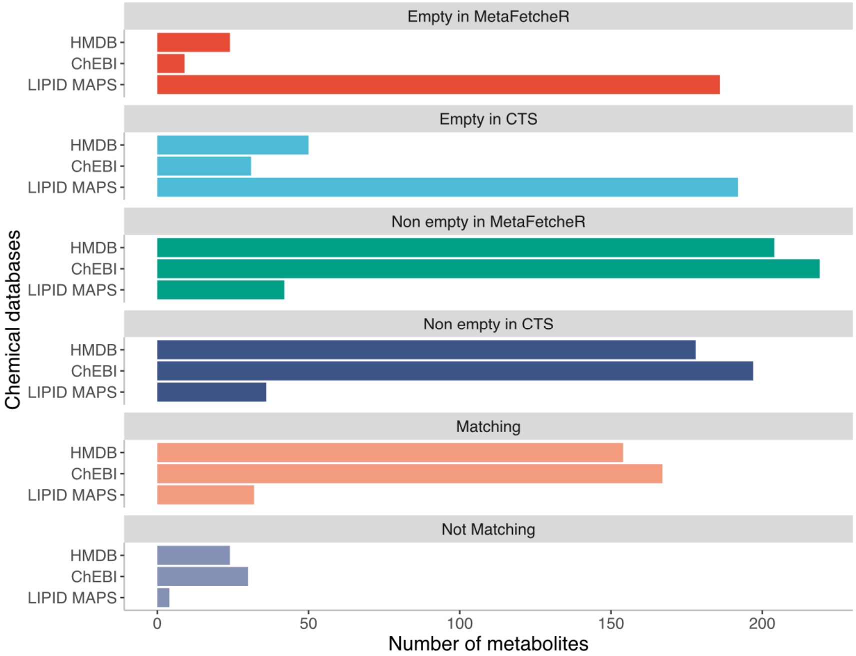

Supplementary Figure S5.Performance comparison based on mapping KEGG identifiers available in Priolo et al [2] dataset to other identifiers. Empty in MetaFetcheR and CTS panels illustrate the number of identifiers that could not be mapped using the respective tool. Non-empty in MetaFetcheR and CTS panels present the number of identifiers that were successfully mapped using the respective tool. Matching panel shows the number of mapped identifiers that agreed between tools. Non-matching panel shows the number of mapped identifiers that were not in agreement between tools. The number of identifiers is shown on the x-axis

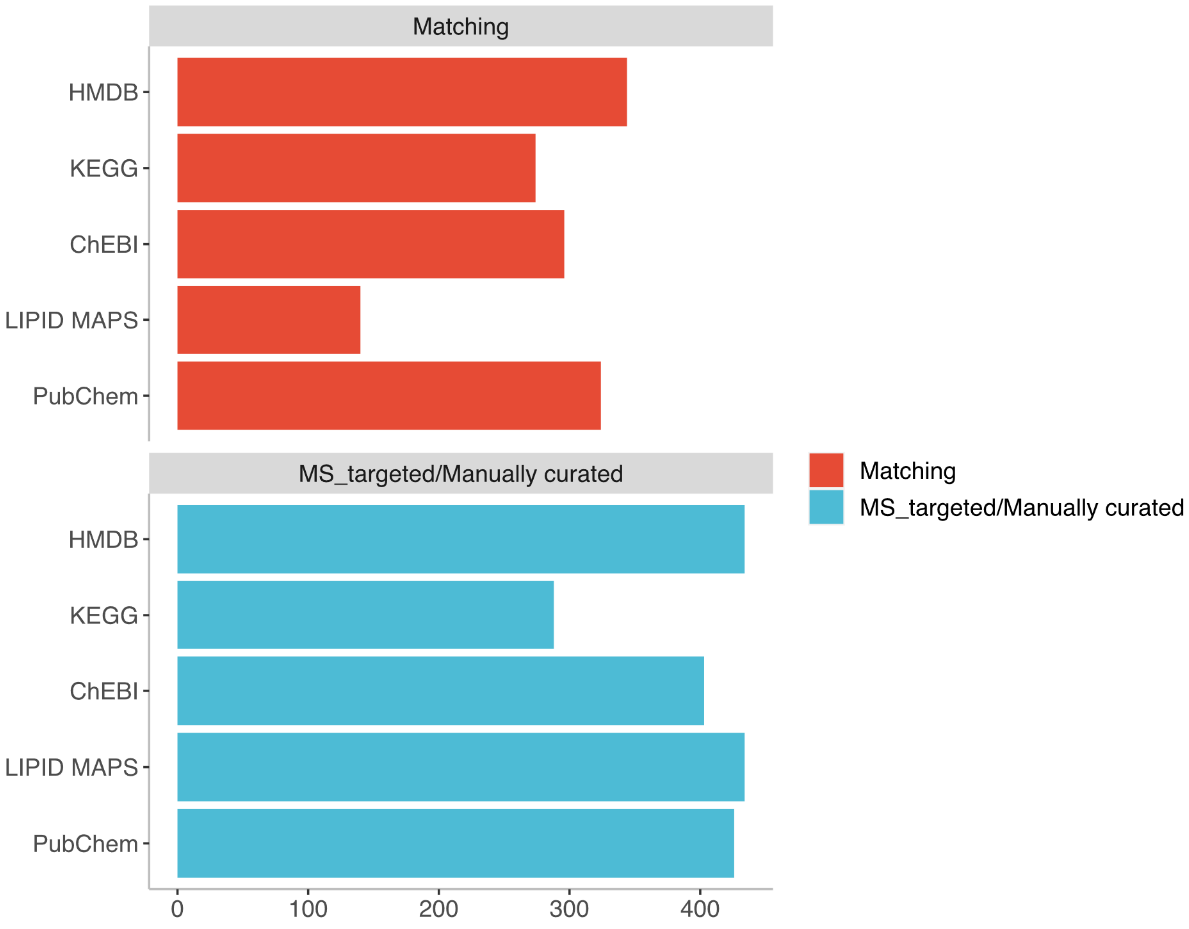

Supplementary Figure S6. Results of comparing MetaFetcheR to MS_targeted with manual curation using Diamanti et al [1] dataset. Red bars represent the number of identifiers mapped by MetaFetcheR that are in agreement with MS_targeted followed by manual curation. Blue bars represent the number of mapped identifiers by MS_targeted followed by manual curation that are in agreement with MetaFetcheR. There is ~80% overlap between MetaFetcheR mapped identifiers and MS_targeted mapped identifiers that have manually been curated.

References

[1] Diamanti K, Cavalli M, Pan G, Pereira MJ, Kumar C, Skrtic S, et al. Intra- and inter-individual metabolic profiling highlights carnitine and lysophosphatidylcholine pathways as key molecular defects in type 2 diabetes. Scientific Reports 2019;9:9653. https://doi.org/10.1038/s41598-019-45906-5.

[2] Priolo C, Pyne S, Rose J, Regan ER, Zadra G, Photopoulos C, et al. AKT1 and MYC Induce Distinctive Metabolic Fingerprints in Human Prostate Cancer. Cancer Res 2014;74:7198–204. https://doi.org/10.1158/0008-5472.CAN-14-1490.

[3] Pang Z, Chong J, Li S, Xia J. MetaboAnalystR 3.0: Toward an Optimized Workflow for Global Metabolomics. Metabolites 2020;10:186. https://doi.org/10.3390/metabo10050186.

[4] Pang Z, Chong J, Zhou G, de Lima Morais DA, Chang L, Barrette M, et al. MetaboAnalyst 5.0: narrowing the gap between raw spectra and functional insights. Nucleic Acids Research 2021;49:W388–96. https://doi.org/10.1093/nar/gkab382.

[5] Wohlgemuth G, Haldiya PK, Willighagen E, Kind T, Fiehn O. The Chemical Translation Service--a web-based tool to improve standardization of metabolomic reports. Bioinformatics 2010;26:2647–8. https://doi.org/10.1093/bioinformatics/btq476.
